# Effectiveness of a learning support program for respiratory physical assessment: A quasi-experimental study

**DOI:** 10.1371/journal.pone.0202998

**Published:** 2018-09-12

**Authors:** Rika Mitoma, Toyoaki Yamauchi

**Affiliations:** 1 Department of Nursing, Graduate School of Health Sciences, Kumamoto University, Kumamoto, Japan; 2 Division of Human Life and Health Sciences, Graduate School of Arts and Sciences, The Open University of Japan, Chiba, Japan; Waseda University, JAPAN

## Abstract

**Background:**

Although the need for physical assessment in home nursing care has been shown, little research has examined the development of visiting nurses’ physical assessment capabilities.

**Objectives:**

This study aimed to evaluate the effectiveness of a learning support program to help working visiting nurses utilize their respiratory physical assessment skills.

**Design:**

This study used a quasi-experimental design of two groups with pre-test, post-test measurements of training and follow-up test 8 weeks practice in their work place after the training.

**Methods:**

57 currently working visiting nurses were assigned to 1 of 2 groups: a control group in which nurses would not receive any special support after respiratory physical assessment training, and an experimental group in which nurses would receive support to utilize the knowledge and skills they had learned during the training.

**Results:**

The average practical examination score was significantly higher after the training. In the experimental group, the average score 8 weeks after training was not significantly higher than that obtained just after training. Further, after 8 weeks, the experimental group’s average score was not significantly higher than that of the control group. Practice evaluation scores obtained by an investigation of daily clinical practice were not significantly higher seventh weeks compared to third weeks after training for either the experimental or control groups. Seventh weeks after training, the average practice evaluation score of the experimental group was significantly higher than that of control groups. The mean learning support program satisfaction score was higher among the experimental group than the control group.

**Conclusions:**

The results suggested that support from others was effective in maintaining motivation for learning and making use in the regular practice of the knowledge and skills acquired in the respiratory physical assessment training.

## Introduction

With medical care scenes shifting from the hospital to the local community, an aging population, and increasingly complex community care management, new medical needs have emerged. It is necessary to redesign education programs to reflect the changing social landscape [1]. More accurate nursing assessments will lead to better quality nursing care; therefore, it is essential to teach physical nursing skills [2]. It is necessary to establish an educational program to improve nurse's physical assessment capabilities, and to evaluate the program.

Recent studies on physical assessment skills have reported that only one-third of the physical assessment skills taught in a basic nursing education curriculum are routinely put into practice [3–6]. Physical assessment skills related to the respiratory system are considered to be among the core physical assessment skills [5]. Osborne et al. found that nurses’ physical assessment core skill sets primarily comprise vital signs [7]. Literature regarding factors influencing nurses’ use of physical assessment skills has identified several constraining factors, including lack of confidence [8, 9], lack of time [3, 8], lack of support from others [9], belief that using the skill is outside the scope of nursing practice [4, 8, 9] and a lack of nursing role models [8]. Douglas et al. [8] reported that experienced registered nurses (RN) may be less influenced by these constraining factors, while less experienced RNs may be more likely to be influenced by a lack of nursing role models. Thus, there are a number of factors that inhibit the use of physical assessment skills, and although these skills are extensively taught in nursing education programs they are not put into practice. In view of this situation, it is necessary to narrow down the content and teach physical assessment skills in such a way that it reduces the constraining factors. Since the less experienced RNs are more easily influenced by such constraining factors, it is particularly important to support them in their practice.

Nurses’ areas of specialty reportedly determine the physical assessment skills they practice [3, 4, 5, 7], and nurses in specialties allowing greater autonomy are more likely to master and practice a broader range of skills. Japanese people’s long life expectancies and low birthrates have led to a rapidly aging population. To cope with this situation, the government introduced a long-term public care insurance system in 2000 [10]. This system provides various home care services, the most important of which is home-visit nursing care, which is the responsibility of “home-visit nurses” (HVNs) [11]. Japan’s HVNs have shown leadership in promoting home care, and their roles have expanded [12]. Home-visit nursing takes place in the convalescents’ private living spaces; as such, HVNs are expected to demonstrate advanced practical skills and exercise autonomy. To this end, it is necessary for nurses to master and practice more advanced physical assessment skills.

As the medical environment changes, so too do the specialized medical education curricula. For nurses, it is essential to stay interested and motivated in continuing education and lifelong learning. Glass [13] found that nurses are more motivated to continue learning when they have a program that is immediately available and encouragement from administrators and colleagues. Duff et al. [14] pointed out that, despite the emphasis on the need for continuing education for nurses, there has been scant empirical evidence of program effectiveness. Scientific and technological progress has broadened awareness of health-related fields. Using simulation-based learning in lieu of clinical practice hours has proven effective among pre-licensure nursing students in the US and UK. In an umbrella review, Cant [15] found that simulation experience supports knowledge acquisition, psychomotor development, self-efficacy, student satisfaction with simulation education, confidence, and critical thinking. In a systematic review, Rutherford-Hemming et al. noted advantages with such simulation; for example, it takes place in a safe and controlled environment, students can repeat an exercise, and students can practice skills on clinical cases for which they lack experience. The review also highlighted disadvantages; i.e., simulation programs fail to reproduce human expressions and communication, and they consume students’ time [16]. In designing simulation programs, it is necessary to use reliable and effective evaluative tools to measure student responses to the simulation. The literature has discussed tools that assess the three domains of learning (cognitive, psychomotor, and affective) simultaneously [17], but the effectiveness and reliability of these tools has not yet been demonstrated.

Kirkpatrick [18] developed a model for assessing training consisting of four levels: reaction, learning, behavior, and results [19]. At Level 1 (reaction), you measure how the trainees react to the training; at Level 2 (learning), you measure what the trainees have learned; at Level 3 (behavior), you evaluate the extent to which the trainees have changed their behaviors and applied what they learned in their job. To determine whether trainees are using the skills they acquired during the training, it is necessary to conduct a follow-up evaluation some months later. At Level 4 (results), you evaluate the final results of the training. Level 4 also includes any outcomes that impact the performance of the relevant organization. As such, Level 4 takes time. The Kirkpatrick Model provides a framework for evaluating the effectiveness of educational simulation programs. Rouse described how the Kirkpatrick Model can be used to determine the effectiveness of courses so that improvements can be made [20]. The Kirkpatrick Model has been used to evaluate simulation-based learning for nurse practitioners.

However, while Levels 1 and 2 are frequently used, Level 3 is seldom used [21, 22], and Level 4 has not been used at all [16, 23]. In a quasi-experimental study, Chavda [24] used Levels 1 and 2 to compare the efficacy of two methods of learning among students. According to Shin, to determine the transfer of learning to practice and outcomes among patients, one has to evaluate the situation in clinical settings. This explains why most of the literature on simulation-based learning for nursing students has focused on student reactions and knowledge acquisition [25]. Some studies reported favorable results in Level 2; namely, that students’ knowledge acquisition improved, but they also noted that knowledge levels declined one to two months after the intervention [26] One post-intervention follow-up found that undergraduate nurses retained their knowledge [27], and another found that their knowledge impacted their workplace practice [28].

In a quasi-experimental study with no control group, Liaw et al. used all four levels of the Kirkpatrick Model to evaluate the effectiveness of web-based simulation in workplace practice with deteriorating ward patients [29]. For Level 1, they used Keller’s model of motivational design [30] to evaluate participants’ motivational reactions to the program; for Level 2, they assessed the participants’ knowledge of clinical deterioration through a questionnaire; for Level 3, they evaluated training transfer at the workplace through a self-reported questionnaire conducted 3–4 months after the intervention; and for Level 4, they examined the frequency at which the participants were detecting deteriorating cases over a 6-month period. We were unable to find any experimental study that examined a program of physical assessment training for HVN and also evaluated whether the training was transferred to the workplace during the course of continuing education.

In this study, we focused on physical assessment skills related to the respiratory system, since these are held to be core physical assessment skills. We developed an education program that trained HVNs in these physical assessment skills and provided them with post-training support. The purpose of this study was to verify the effectiveness of this program using the Kirkpatrick Model.

## Methods

### Design

This study used a quasi-experimental design in which HVNs were assigned to groups that either received respiratory physical assessment training and subsequent support for workplace technique utilization for 8 weeks after the training (experimental group), or received respiratory physical assessment training but not the subsequent support (control group). The two groups were then compared.

### Research hypothesis

1. Respiratory physical assessment knowledge and skills are acquired through respiratory physical assessment training.
2. Support provided by the researchers after training will increase the knowledge and skills acquired during training.
3. Use of the knowledge and skills acquired during training in their workplace will be increased by the support provided by the researchers after training.
4. Support provided by the researchers after training will raise satisfaction with the program.

### Subjects

#### Program participants

The study participants were currently employed as HVNs who had less than 10 years’ experience; thus, they had not reached the “expert” level of the five-step Patricia Benner’s theoretical model of novice to expert [31]. They had HVN experience of more than 1 year, except for the novices, because most of the HVNs had gained prior clinical practice experience.

Written requests for study participation were mailed to nursing supervisors at HVN stations; the supervisors then gave the written participation requests and applications to the HVNs who met the conditions. HVNs who met the conditions and were willing to participate in the study then returned the applications by fax or email. The participants indicated their consent to participate in the study after receiving a written briefing before the respiratory physical assessment training.

#### Allocation

Sequential assignment using the minimization method was performed while striving for balance in overall numbers. Allocation adjustment factors included possible confounding factors, such as practical test score on the test after training, motivation for participation, and visited patient information. The allocation was done by a research assistant who received instruction from a specialist in epidemiological and biological statistics.

### Learning support program development

Using the system approach of instructional design [32], which is based on situational learning theory [33] and a learning transfer model [34], a respiratory physical assessment learning support program was developed for HVNs with limited experience.

#### Formulation of educational content and methods

Educational content and methods, evaluation indices, and evaluation methods were formulated based on the literature and the results of a preliminary study that used interviews with HVNs who had completed a master’s course in community nursing or who were certified in home-visit nursing, had at least 5 years of experience as HVNs, and were currently engaged in home-visit nursing.

The educational content was limited to respiratory, because HVNs have a great need for breath sound auscultation skills, and such techniques are used frequently in HVN practice. The training consisted of lectures using audiovisual teaching materials, exercises using a simulator, reasoning exercises using cases in which symptoms appeared, and practices on respiratory physical assessment, so the learned topics could be applied in HVN activities.

Application of knowledge and skills acquired during training to their workplace practices requires workplace training; thus, the practice in their workplace for 8 weeks to apply the acquired skills to the workplace after training was included in this program. A control group would not receive any special support after respiratory physical assessment training, and an experimental group would receive support to utilize the knowledge and skills they had learned during the training.

#### Evaluation methods

The four-level evaluation by Kirkpatrick was used to assess the learning support program.

Level 2 evaluates the level of achievement of the training targets; this evaluation was done with a test on physical assessment knowledge and skills related to the training lectures and exercises. The practical test consisted of task1, task 2, and skill. The task 1 test consisted of 25 items of respiratory physical assessment skills, and the score was 0–52 points. The task test 2 consisted of 5 cases of reasoning, and the score range was 0–25 points. The skill test consisted of 12 items of respiratory information collection, and the score was 0–12 points. The evaluation of level 2 was conducted before training, after training, and 8 weeks after training. A questionnaire on demographic data of participant was conducted before training.

Level 3 evaluates whether knowledge and skills gained during training can be applied in professional practice. A survey of the state of implementation in their workplace of sixteen items acquired in the training and the reasons for "implemented" and "not implemented" was evaluated for each one week in third week and seventh week after training. The score range was 0–16 points each one patient.

Level 1 was evaluated based on the degree of satisfaction with the learning support program. The questionnaire consisted of 10 items related to the content and method of the learning support program using a 5-point Likert response scale and open-ended questions about the learning support program. The score range was 10–50 points. All participants were asked to complete the questionnaire after the completion of the learning support program.

#### Investigation of the validity of the educational content and methods, and the evaluation methods

The validity of the developed educational content, educational methods, and assessment methods developed repeatedly investigated and determined by a co-researcher who was accomplished in physical assessment education and research, as well as by a certified HVNs who participated in the study. In exercises using physical examination skills and case examples, the eight certified HVNs acted as instructors.

#### Investigation of the reliability of instruction and assessment methods

So the eight certified HVNs could teach the techniques in the exercises, they confirmed the current status of physical examination implementation before providing instruction. The evaluation criteria for practical examination were discussed and were repeatedly evaluated until the κ coefficient for agreement between the researchers and assessors reached ≥0.7. The reliability of the questionnaire was investigated using a repeat test method with the participants’ consent.

### Data analysis

Student’s t-test and Fisher’s direct test were used for the comparisons between the subject characteristics and the visited patient information of the two groups. Student’s paired t-tests were used to determine differences between the two independent groups (i.e., the experimental and control groups) in terms of total score on the respiratory physical assessment practical test, practical test scores by time course, and satisfaction with the training. A χ^2^, or Fisher’s direct, test was performed for presence or absence of implementation, and the Mann-Whitney test or Wilcoxon’s signed rank test was performed for selection between groups with and without implementation.

SPSS version 18.0 J for Windows statistical software was used for the analysis.

### Ethical considerations

This study was approved by the ethics committee of St. Luke's University. The nurses indicated their consent to participate in the study after receiving a written briefing. The briefing informed the participants that:

- Participation was entirely voluntary
- The questionnaire contained no items that could be used to identify them
- They were free to withdraw at any time
- The results would only be used on an aggregated basis
- The decision to participate in the study would not influence their participation in the learning support program

The questionnaire was designed to ensure that individual respondents could not be identified. We also asked the nurses to indicate their ID number on each questionnaire so that we could match the data. There was no way to infer identity from the number given.

## Results

### Participant characteristics

Fifty-seven HVNs who consented to the study participated in the training. The participants were randomized using a minimization method: 29 and 28 were assigned to the experimental and control groups, respectively. Two and three participants from the experimental and control groups dropped out after the training, respectively. Accordingly, there were 27 and 25 participants for the practical test 8 weeks after the training in the experimental and control groups, respectively. There were no statistically significant differences in the participant characteristics of the experimental and control groups; thus, the groups were considered nearly homogenous (Table 1).

**Table 1:** Participant characteristics.

Characteristic	Exp. group (n = 29)	Control group (n = 28)	*p* value
	Mean ± SD	Mean ± SD	
Nursing experience (years)	12.64±5.63	10.92±4.79	0.22
Home visiting nursing experience (years)	2.39±1.37	2.30±1.51	0.81
Motivation for participation	16.31±1.51	15.96±1.81	0.44
Workplace support status	2.69±0.97	2.61±0.69	0.71
Visited patient characteristics			
Number of visited patients	16.41±5.37	14.32±5.70	0.16
Patient age	74.84±9.67	75.96±5.65	0.59
Care need level	4.62±1.22	4.54±0.95	0.78
Respiratory status	0.95±0.53	0.88±0.48	0.61
Have taken physical assessment course	No. people (%)	No. people (%)	
Yes	13 (44.8%)	15 (53.6%)	0.51
No	16 (55.2%)	13 (46.4%)	

Visited patient characteristic: t-test; Have taken physical assessment course only: χ^2^ test

### Analysis results for the hypothesis

#### Hypothesis 1

The differences in the mean values of the practical tests before and after training were 17.14 (SD 7.41) and 29.75 (SD 6.87), respectively. The mean practical test score after the training was significantly different than that before the training (t (56) = −13.84, *p* < 0.01 [Table 2]).

**Table 2:** Practical test: Comparison of mean scores before and after training.

Practical test	Before training (n = 57)	After training (n = 57)	t value	Degrees of freedom	*p* value	
	Mean ± SD	Mean ± SD				
Task 1	5.32±3.25	8.23±3.95	-5.44	56	< 0.01	**
Task 2	5.75±4.04	12.96±4.29	-11.47	56	< 0.01	**
Skill	6.07±1.94	8.56±1.79	-8.38	56	< 0.01	**
Total	17.14±7.41	29.75±6.87	-13.84	56	< 0.01	**

Practical test: t-test

** p < 0.01

#### Hypothesis 2

In the experimental group, the score 8 weeks after the training was 30.76 (SD 7.16), and the score soon after the training was 29.24 (SD 7.95). No significant increase was evident (t (28) = −1.16, *p* = 0.254). In the practical test 8 weeks after the training, the experimental group’s score was 30.76 (SD 7.16), and that of the control group was 31.36 (SD 6.72). Again, no significant difference was apparent (t (55) = -0.33, *p* = 0.746) (Table 3).

**Table 3:** Practical test: Comparison of mean scores between two groups directly and 8 weeks after training.

Practical test		Ex. group (n = 29)	Control group (n = 28)	t value	Degrees of freedom	*p* value
		Mean ± SD	Mean ± SD			
After training	Task 1	8.41±4.46	8.04±3.42	0.36	55	0.721
	Task 2	12.69±4.91	13.25±3.63	-0.49	55	0.627
	Skill	8.14±1.98	9.00±1.49	-1.85	55	0.069
	Total	29.24±7.95	30.29±5.63	-0.57	55	0.571
8 weeks after training	Task 1	9.76±4.76	10.36±4.07	-0.51	55	0.612
	Task 2	12.93±4.39	12.75±3.82	0.17	55	0.869
	Skill	8.07±1.62	8.25±1.69	-0.41	55	0.682
	Total	30.76±7.16	31.36±6.72	-0.33	55	0.746

Practical test: t test

#### Hypothesis 3

In the survey of in their workplace practices after the training, the scores in the third week were 10.53 (SD 2.35) and 9.09 (SD 2.84) for the experimental and control groups, respectively, and the difference was significant (t (51) = 2.02, *p* = 0.049). The scores in the seventh week after the training were 10.71 (SD 2.60) and 9.28 (SD 2.55) for the experimental and control groups, respectively, and a significant difference was seen (t (51) = 2.01, *p* = 0.049). However, in the experimental group, the practice evaluation score in the seventh week after training (10.71, SD 2.60) was not higher than that in the third week (10.53, SD 2.35; t (26) = −0.68, *p* = 0.502), and this result differed from that of the control group. In the seventh week after training, the experimental group implemented respiratory physical assessment significantly more often than did the control group (Table 4).

**Table 4:** Between-group comparison of "respiratory physical assessment implementation".

Respiratory physical assessment item	Exp. group (n = 29)	Control group (n = 28)	*p* value	
3^rd^ week Respiratory physical assessment implementation score	Mean ± SD	Mean ± SD		
	10.53±2.35	9.09±2.84	0.049	*
Total no. of visited patients	377	370		
Respiratory physical assessment item	Exp. group (n = 27)	Control group (n = 26)	*p* value	
7^th^ week Respiratory physical assessment implementation score	Mean ± SD	Mean ± SD		
	10.71±2.60	9.28±2.55	0.049	*
Total no. of visited patients	371	328		

Respiratory physical assessment implementation score: t-test

* p < 0.05

The experimental group performed assessments more frequently than did the control group, except for the “auscultation of respiratory sounds,” which both groups performed equally. The performance survey given after training revealed that the experimental and control groups lacked confidence in their skills, judgments, and explanations both during and after performing the physical assessment of the respiratory system. However, fewer nurses responded that they “lack confidence” and “lack knowledge” after receiving support. The participants reported that recording their practice became a reflection of their performance.

#### Hypothesis 4

As shown in Table 5, the mean satisfaction levels were 39.30 (SD 4.61) and 34.56 (SD 5.08) for the experimental and control groups, respectively; the level was significantly higher in the experimental group (t(50) = 3.56, *p* = 0.001). Thus, Hypothesis 4 was supported.

The experimental group’s satisfaction with “the learning support program,” “intelligibility of learning materials,” “received support,” and “support methodology” were significantly higher than that of the control group.

These participants also gave positive feedback about the program in their free descriptive responses, examples of which include the following: “the training content reflected actual practice,” “I improved my knowledge and skills,” “I can continue learning,” “during the follow-up test, knowledge could be shared,” and “by recording how often I use the skills, I was able to reflect on my past practice.”

**Table 5:** Between-group comparisons of "satisfaction with learning support program".

Participant satisfaction		Exp. group (n = 29)		Control group (n = 28)		t value	Degrees of freedom	*p* value	
		Mean ± SD	Range	Mean ± SD	Range				
1	Satisfaction with learning support program	4.33±0.56	3–5	3.76±0.83	2–5	2.95	50	< 0.01	**
2	Utilization of visiting nurse activities	4.07±0.92	2–5	3.88±0.83	3–5	0.79	50	0.429	
3	Ease of understanding of teaching materials	4.38±0.57	3–5	3.92±0.86	2–5	2.28	49	0.027	*
4	No. of hours of training	3.48±0.70	2–5	3.17±0.82	2–4	1.48	49	0.145	
5	Learning support program period	3.81±0.79	2–5	3.52±0.77	2–5	1.36	50	0.179	
6	Receive support	3.85±0.77	3–5	2.96±0.89	1–5	3.88	50	< 0.01	**
7	Support method	4.15±0.60	3–5	3.43±0.73	2–5	3.79	48	< 0.01	**
8	Promotion of voluntary learning	3.74±0.76	3–5	3.36±0.70	2–5	1.87	50	0.068	
9	Improved motivation for respiratory physical assessment	3.78±0.97	2–5	3.36±0.58	2–5	1.72	50	0.092	
10	Eagerness for other than respiratory physical assessment	3.86±1.03	2–5	3.60±0.82	2–5	0.97	50	0.335	
Total		39.30±4.6		34.56±5.08		3.53	50	< 0.01	**

Satisfaction: t-test

** p < 0.01

* p < 0.05

## Discussion

### Validity of the learning support program

In this study, we focused on physical assessment skills related to the respiratory system, since these are held to be core physical assessment skills. We developed an education program that trains HVNs in these physical assessment skills and provides post-training support. We then evaluated the effectiveness of this program using the Kirkpatrick Model. Providing post-training support led to improved satisfaction with the program (Level 1). Participants mastered knowledge and skills for respiratory assessment as a result of the program (Level 2), and they retained these skills and this knowledge at 8 weeks after training. However, the results did not demonstrate that post-training support had a beneficial effect on acquired knowledge and skills. HVNs who received post-training support were more likely to employ the respiratory assessment skills they learned during the training in their home-visit practice (Level 3). Thus, our program succeeded in transferring the learning (respiratory assessment skills) to HVN practice. The results also indicated that the provision of post-training support facilitated this transfer.

Existing studies regarding the efficacy of using simulation programs assessed the learning outcomes of such interventions on Levels 1 and 2 of the Kirkpatrick Model, and they reported favorable results for knowledge acquisition and student satisfaction [16, 23]. Our study echoes these findings. Other studies reported that knowledge initially improved as a result of the intervention but declined 1–2 months later [26]. In the test 8 weeks after training, we confirmed that the participants retained the knowledge and skills they acquired during the training. The results of the follow-up test did not significantly differ between the experimental and control groups, so we were unable to demonstrate whether providing post-training support was efficacious in this respect.

The training, which both groups received, consisted of lectures using audiovisual materials, simulator-based exercises, and reasoning exercises based on cases featuring certain symptoms. According to Liaw [29], simulation-based training that closely mirrors real-life settings creates a learning environment that reflects the methods in which knowledge and skills are actually employed. Such authenticity is an important facilitator of the transfer of learning [29]. We aimed to ascertain the extent to which the participants used their acquired knowledge and skills within the workplace. Accordingly, we asked the participants, including those in the control group, to fill out forms third and seventh weeks after the training, upon which they were to state the extent to which they were using the knowledge and skills. According to the participants’ feedback, in recording the extent of their use of knowledge and skills they were able to reflect upon their past practice. Thus, the requirement to report this information at third and seventh weeks evidently had an intervention effect in the control group too. This might explain why we did not observe any significant difference between the groups 8 weeks after intervention.

The analysis of the participants’ post-training use of skills indicated that they lacked confidence in their skills, and in their abilities to make decisions and give explanations, both during and after training. Lack of confidence [8, 9] and inadequate knowledge are barriers to using physical assessment skills. In our study, however, “I lack knowledge about this skill” and “I do not feel confident about performing this skill” were cited less often as reasons for not using a skill after the training compared to before. The post-training support at 8 weeks consisted only of one interview, and this might not have led to a thorough exercising of the skills. However, the support might have improved the participants’ confidence and knowledge by allowing them to discuss and clarify the areas that they did not fully master during the training. This, in turn, may have empowered them to start using skills they previously felt incapable of using.

Literature regarding factors that influence nurses’ use of physical assessment skills has identified several constraining factors, including lack of confidence [8,9], lack of time [3, 8], lack of support from others [9], belief that using the skill is outside the scope of nursing practice [4, 8, 9], and a lack of nursing role models [8]. Because HVNs work in patients’ homes by themselves, they would benefit greatly from opportunities to evaluate whether their self-appraisals are appropriate, and to discuss problems they experience in their practice. However, nurses may miss such opportunities because of their busy schedules. It is therefore essential that supporting parties proactively encourage the nurses to avail themselves of such opportunities. To this end, it is necessary to develop a community of practice conducive to mutual learning—one in which managers, senior staff, and colleagues exchange knowledge and information through legitimate peripheral participation [33].

The participants who received post-training support were highly satisfied with the program. These participants also gave positive feedback about the program in their free descriptive responses, examples of which include the following: “the training content reflected actual practice,” “I improved my knowledge and skills,” “I can continue learning,” “during the follow-up test, knowledge could be shared,” and “by recording how often I use the skills, I was able to reflect on my past practice.” Confidence and satisfaction are two components in Keller’s ARCS (attention, relevance, confidence, satisfaction) model [30]—a model of instructional design for raising motivation. According to this model, these increase learner motivation. Thus, the feedback suggests that support from others helped the HVNs to maintain their motivation, gain confidence, and employ the respiratory assessment skills and knowledge they acquired during training.

### Limitations

The program we developed pertains only to respiratory assessment skills, and we only tested it on HVNs with fewer than five years’ experience. Our sample was too small, and the learning support period too short, to fully demonstrate the effectiveness of the program. It is necessary to conduct further research with a larger sample of HVNs and greater learning support follow-up. Moreover, to evaluate the outcomes among patients, it will be necessary to analyze core physical assessment skills besides those related to the respiratory system.

## Conclusion

In this study, we focused on physical assessment skills related to the respiratory system, since these are eheld to be core physical assessment skills. We developed an education program that trains HVNs in these physical assessment skills and provides post-training support to help transfer these skills to the workplace. The results indicated that support from others helped the learners maintain their motivation, gain confidence, retain the respiratory assessment skills and knowledge they acquired in the training, and enabled them to transfer these skills and that knowledge to the workplace. It is necessary to provide ongoing learner support and additionally evaluate outcomes among patients (Level 4 of the Kirkpatrick Model).

## References

[1] Tanner CA. Transforming prelicensure nursing education: preparing the new nurse to meet emerging health care needs. Nurs Educ Perspect. 2010; 31(6): 347–353. 21280438

[2] Tuzer H, Dinc L, Elcin M. The effects of using high-fidelity simulators and standardized patients on the thorax, lung, and cardiac examination skills of undergraduate nursing students. Nurse Educ Today. 2016; 45: 120–125. 10.1016/j.nedt.2016.07.002 27449150

[3] Birks M, Cant R, James A, Chung C, Davis J. The use of physical assessment skills by registered nurses in Australia: issues for nursing education. Collegian. 2013; 20(1): 27–33. 23678781 10.1016/j.colegn.2012.02.004

[4] Cicolini G, Tomietto M, Simonetti V, Comparcini D, Flacco ME, Carvello M, et al Physical assessment techniques performed by Italian registered nurses: a quantitative survey. J Clin Nurs. 2015; 24(23–24): 3700–37066. 10.1111/jocn.12997 26419409

[5] Giddens JF. A survey of physical assessment techniques performed by RNs: lessons for nursing education. J Nurs Educ. 2007; 46(2): 83–87. 17315568 10.3928/01484834-20070201-09

[6] Secrest JA, Norwood BR, duMont PM. Physical assessment skills: a descriptive study of what is taught and what is practiced. J Prof Nurs. 2005; 21(2): 114–118. 15806509 10.1016/j.profnurs.2005.01.004

[7] Osborne S, Douglas C, Reid C, Jones L, Gardner G, Council RPAR. The primacy of vital signs—acute care nurses' and midwives' use of physical assessment skills: a cross sectional study. Int J Nurs Stud. 2015; 52(5): 951–962. 10.1016/j.ijnurstu.2015.01.014 25704371

[8] Douglas C, Osborne S, Reid C, Batch M, Hollingdrake O, Gardner G, et al What factors influence nurses' assessment practices? Development of the Barriers to Nurses' Use of Physical Assessment Scale. J Adv Nurs. 2014; 70(11): 2683–2694. 10.1111/jan.12408 24702032

[9] McElhinney E. Factors which influence nurse practitioners ability to carry out physical examination skills in the clinical area after a degree level module—an electronic Delphi study. J Clin Nurs. 2010; 19(21–22): 3177–3187. 10.1111/j.1365-2702.2010.03304.x 21040021

[10] Tamiya N, Noguchi H, Nishi A, Reich MR, Ikegami N, Hashimoto H, et al Population ageing and wellbeing: lessons from Japan's long-term care insurance policy. Lancet. 2011; 378(9797): 1183–1192. 10.1016/S0140-6736(11)61176-8 21885099

[11] Asahara K, Ono W, Kobayashi M, Omori J, Momose Y, Todome H, et al Ethical issues in practice: a survey of home-visiting nurses in Japan. Jpn J Nurs Sci. 2013; 10(1): 98–108. 10.1111/j.1742-7924.2012.00216.x 23735094

[12] Murashima S, Nagata S, Magilvy JK, Fukui S, Kayama M. Home care nursing in Japan: a challenge for providing good care at home. Public Health Nurs. 2002; 19(2): 94–103. 11860594 10.1046/j.1525-1446.2002.19204.x

[13] Glass JC, Todd-Atkinson S. Continuing education needs of nurses employed in nursing facilities. J Contin Educ Nurs. 1999; 30(5): 219–228. 10808838 10.3928/0022-0124-19990901-07

[14] Duff B, Gardner G, Osborne S. An integrated educational model for continuing nurse education. Nurse Educ Today. 2014; 34(1): 104–111. 10.1016/j.nedt.2012.11.022 23266059

[15] Cant RP, Cooper SJ. The value of simulation-based learning in pre-licensure nurse education: a state-of-the-art review and meta-analysis. Nurse Educ Pract. 2017; 27: 45–62. 10.1016/j.nepr.2017.08.012 28843948

[16] Rutherford-Hemming T, Nye C, Coram C. Using simulation for clinical practice hours in nurse practitioner education in the United States: a systematic review. Nurse Educ Today. 2016; 37: 128–35. 10.1016/j.nedt.2015.11.006 26608389

[17] Radhakrishnan K, Roche JP, Cunningham H. Measuring clinical practice parameters with human patient simulation: a pilot study. Int J Nurs Educ Scholarsh. 2007; 4: Article 8. 10.2202/1548-923X.1307 17402934

[18] Kirkpatrick DL. Evaluating training programs—the four levels. San Francisco: Berrett-Koehler; 1998.

[19] Reiser RA, Dempsey JJ. Trends and issues in instructional design and technology. 3^rd^ Edition Boston: Pearson; 2012.

[20] Rouse DN. Employing Kirkpatrick's evaluation framework to determine the effectiveness of health information management courses and programs. Perspect Health Inf Manag. 2011; 8: 1c. PMC3070232 21464860

[21] Curtis JR, Back AL, Ford DW, Downey L, Shannon SE, Doorenbos AZ, et al Effect of communication skills training for residents and nurse practitioners on quality of communication with patients with serious illness: a randomized trial. JAMA. 2013; 310(21): 2271–2281. 10.1001/jama.2013.282081 24302090 PMC4310457

[22] Rutherford-Hemming T. Learning in simulated environments: effect on learning transfer and clinical skill acquisition in nurse practitioner students. J Nurs Educ. 2012; 51(7): 403–406. 10.3928/01484834-20120427-04 22533500

[23] Warren JN, Luctkar-Flude M, Godfrey C, Lukewich J. A systematic review of the effectiveness of simulation-based education on satisfaction and learning outcomes in nurse practitioner programs. Nurse Educ Today. 2016; 46: 99–108. 10.1016/j.nedt.2016.08.023 27621199

[24] Chavda P, Pandya C, Solanki D, Dindod S. Is "modular" the way to go for small group learning in community medicine in undergraduate clinical postings? Int J Appl Basic Med Res. 2016; 6(3): 211–214. 10.4103/2229-516X.186970 27563590 PMC4979306

[25] Shin S, Park JH, Kim JH. Effectiveness of patient simulation in nursing education: meta-analysis. Nurse Educ Today. 2015; 35(1): 176–182. 10.1016/j.nedt.2014.09.009 25459172

[26] Scherer YK, Bruce SA, Runkawatt V. A comparison of clinical simulation and case study presentation on nurse practitioner students' knowledge and confidence in managing a cardiac event. Int J Nurs Educ Scholarsh. 2007; 4: Article 22. 10.2202/1548-923X.1502 18052920

[27] Hart PL, Brannan JD, Long JM, Maguire MB, Brooks BK, Robley LR. Effectiveness of a structured curriculum focused on recognition and response to acute patient deterioration in an undergraduate BSN program. Nurse Educ Pract. 2014; 14(1): 30–36. 10.1016/j.nepr.2013.06.010 23867283

[28] Gordon CJ, Frotjold A, Fethney J, Green J, Hardy J, Maw M, et al The effectiveness of simulation-based blood pressure training in preregistration nursing students. Simul Healthc. 2013; 8(5): 335–340. 10.1097/SIH.0b013e3182a15fa7 24061336

[29] Liaw SY, Wong LF, Lim EY, Ang SB, Mujumdar S, Ho JT, et al Effectiveness of a web-based simulation in improving nurses' workplace practice with deteriorating ward patients: a pre- and post-intervention study. J Med Internet Res. 2016; 18(2): e37 10.2196/jmir.5294 26895723 PMC4779162

[30] Keller JM. Motivational design for learning and performance: the ARCS model approach. New York: Springer; 2010.

[31] Benner B. From Novice to Expert: Excellence and Power in Clinical Nursing Practice, Commemorative Edition. New Jersey: Prentice Hall; 2001.

[32] Dick W, Carey L, Carey OJ. Systematic design of instruction. 5th ed. Boston, MA: Allyn & Bacon; 2001.

[33] Lave J, Wenger E. Situated learning: legitimate peripheral participation. Cambridge: Cambridge University Press; 1991.

[34] Holton EF. The flawed four-level evaluation model. Hum Res Dev Q. 1996; 7(1): 5–21. 10.1002/hrdq.3920070103

